# Topical Simvastatin as Host-Directed Therapy against Severity of Cutaneous Leishmaniasis in Mice

**DOI:** 10.1038/srep33458

**Published:** 2016-09-16

**Authors:** Suraj P. Parihar, Mary-Anne Hartley, Ramona Hurdayal, Reto Guler, Frank Brombacher

**Affiliations:** 1 International Centre for Genetic Engineering and Biotechnology (ICGEB), Cape Town Component, Cape Town, South Africa; 2 Institute of Infectious Diseases and Molecular Medicine (IDM), Division of Immunology and South African Medical Research Council (SAMRC) Immunology of Infectious Diseases, Faculty of Health Sciences, University of Cape Town, Anzio Road, Observatory 7925, Cape Town, South Africa; 3 Department of Biochemistry, University of Lausanne, Chemin des Boveresses 155, Epalinges, CH1066, Switzerland; 4 Department of Molecular and Cell Biology, Faculty of Science, University of Cape Town, Rondebosch-7701, Cape Town, South Africa

## Abstract

We recently demonstrated that statins mediate protection against intracellular pathogens, *Mycobacterium tuberculosis* and *Listeria monocytogenes* in mice. Here, we investigated the immunomodulatory potential of simvastatin as a topical or systemic host-directed drug therapy in controlling inflammatory responses in an experimental mouse model of cutaneous leishmaniasis caused by *Leishmania major* (LV39). In an ear infection model, topical application of simvastatin directly on established lesions significantly reduced severity of the disease reflected by ear lesion size and ulceration. The host protective effect was further accompanied by decreased parasite burden in the ear and draining lymph nodes in both BALB/c and C57BL/6 mice. Pre-treatment of these mice on a low-fat cholesterol diet and systemic simvastatin also reduced footpad swelling, as well as parasite burdens and ulceration/necrosis in the more robust footpad infection model, demonstrating the prophylactic potential of simvastatin for cutaneous leishmaniasis. Mechanistically, following *L. major* infection, simvastatin-treated primary macrophages responded with significantly reduced cholesterol levels and increased production of hydrogen peroxide. Furthermore, simvastatin-treated macrophages displayed enhanced phagosome maturation, as revealed by increased LAMP-3 expression in fluorescent microscopy and Western blot analysis. These findings demonstrate that simvastatin treatment enhances host protection against *L. major* by increasing macrophage phagosome maturation and killing effector functions.

Leishmaniasis is a neglected human parasitic disease of the tropic. A haematophagous sand fly vectors the *Leishmania* parasite, and its numerous species give rise to a variety of clinical manifestations, ranging from localised, disfiguring inflammatory skin lesions to fatal visceral forms. Collectively, over 1.3 million people are infected worldwide^1^. To date, there are no effective vaccines and current first-line therapies are based on an antiquated arsenal of pathogen-directed drugs, such as pentavalent antimonials. These require long term intravenous therapy as well as monitoring for toxic side-effects^2^. Hence, there is a need for adjunctive compounds, which may improve the efficacy and longevity of existing anti-leishmanial drugs or control inflammatory pathology of the host responses^3^.

Host-directed immunotherapeutic have the major advantage of reducing the potential emergence of drug-resistance^4^ and may also interfere with the complex of immune evasion which *Leishmania* parasites has developed in order to promote its survival within the phagolysosome of host macrophages. One such evasion mechanism is the parasite’s ability to reduce macrophage activation by manipulating membrane cholesterol in host cells^5^. Statins are widely used cholesterol-lowering drugs, which target the key rate-limiting enzyme of the cholesterol biosynthesis pathway, hydroxy-3-methylglutaryl coenzyme A (HMG-CoA) reductase^6^. Statins are reported to exert pleiotropic immunomodulatory effects independent of their signature cholesterol-lowering properties^7^,^8^. For example, statins influence anti-inflammatory activity by decreasing MHC-II-mediated T-cell activation^9^. In addition, statin therapy has been associated with reduced mortality in diseases that induce severe hyper-inflammation, such as bacteraemia^10^,^11^ and promotes a protective response against parasitic diseases such as *Toxoplasma gondii*^12^. Previously, we demonstrated that simvastatin has a beneficial effect on the prevention of murine listeriosis^13^ and tuberculosis in both mice and humans^14^. On the other hand, Contrasting reports have shown cholesterol depletion (or statin treatment) to be either beneficial^5^,^15^,^16^ or detrimental^17^,^18^ to the host during *Leishmania* infection.

In this study, we investigated the effect of simvastatin treatment on the pathogenesis of cutaneous leishmaniasis caused by *Leishmania. major* LV39 parasites. We show a novel therapeutic potential for a topical application of simvastatin that reduces tissue damage and parasite burden in lesions caused by *L. major.* In addition, simvastatin also displayed host protective effects when explored for its prophylactic potential, which reduced footpad swellings and parasite burdens in mice. Mechanistically, pre-treatment of primary macrophages with simvastatin resulted in increased production of hydrogen peroxide and phagosome maturation, leading to enhanced killing effector functions.

## Results

### Topical application of simvastatin on ear lesions caused by *Leishmania major* is therapeutic in both BALB/c and C57BL/6 mice

**Figure 1:**
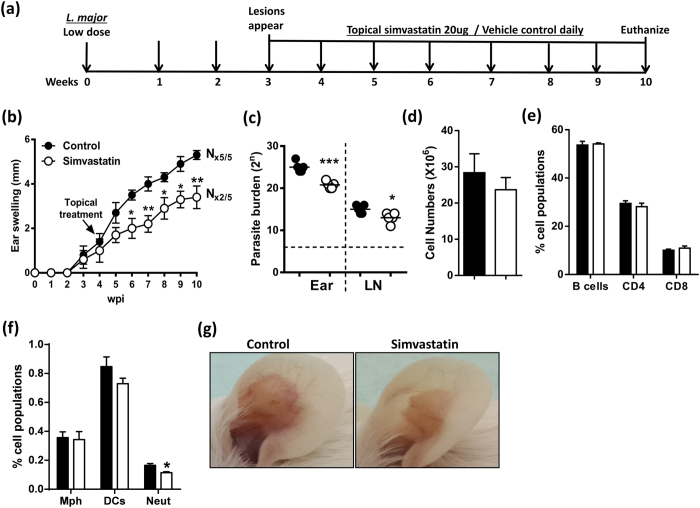
Topical application of simvastatin improves control of *Leishmania major* at low dose infection in BALB/c mice. (**a**) BALB/c mice were infected subcutaneously in the ear dermis with 1 × 10^3^ stationary phase *L. major* LV39 (MRHO/SV/59/P) promastigotes. On the appearance of lesions, ears were treated daily for 7 weeks with topically applied simvastatin (or vehicle control) as shown in the layout. (**b**) Ear swelling was measured weekly. (**c**) Mice were sacrificed to measure parasite burden in the ear dermis and in cervical lymph nodes by limiting dilution assay after 10 weeks of infection. (**d**) Cell numbers recovered from cervical lymph nodes. (**e**) Percentages of lymphocyte populations and (**f**) myeloid cells. The surface markers used to determine the leukocyte phenotypes are as follows; B cells = CD19^+^ CD3^−^, CD4 = CD3^+^ CD4^+^, CD8 = CD3^+^ CD8^+^, Mphs = CD11b^+^ MHCII^+^ CD11c^−^, DCs = CD11c^+^ MHCII^+^ and Neutrophils = Gr1^+^ CD11c^−^. (**g**) Photographs of simvastatin-treated mice displaying reduced lesion size and inflammation. Results are the mean ± SEM of n = 5 mice/group from one experiment, where N denotes the necrotic ear lesions. Statistical analysis was performed defining differences to vehicle treated control mice as significant (*p < 0.05; **p < 0.01; ***p < 0.001).

**Figure 2:**
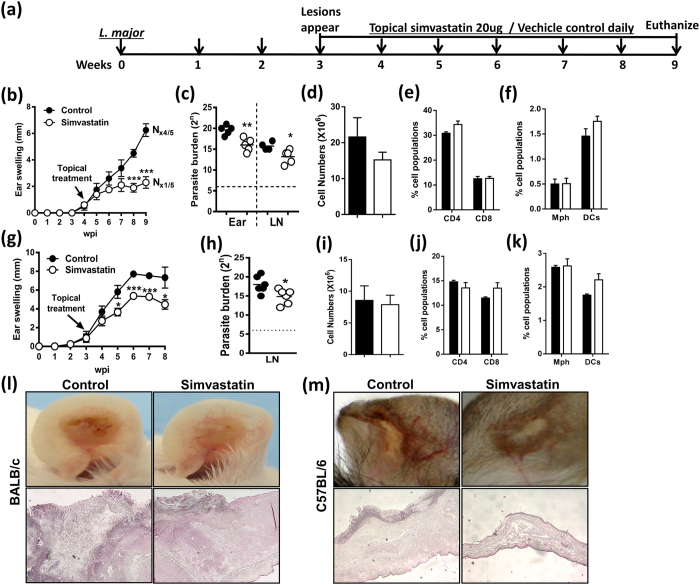
Topical application of simvastatin increases host protection against 10-fold high dose *L. major* infection in both BALB/c and C57BL/6 mice. (**a**) BALB/c and C57BL/6 mice were infected subcutaneously in the dermis of the ear with high dose (1 × 10^4^) stationary phase *L. major* LV39 promastigotes. Following the eruption of lesions, ears were treated daily for 6 weeks (BALB/c) and 5 weeks (C57BL/6) with topically applied simvastatin (or vehicle control) as shown in the layout. (**b**) BALB/c ear swelling was measured weekly. (**c**) Parasite burden in ear and cervical lymph nodes was determined by limiting dilution assay after 9 weeks of infection. (**d**) Cell numbers recovered and (**e,f**) the percentage of immune cell populations in cervical lymph nodes. (**g**) C57BL/6 ear swelling and (**h**) parasite burden in lymph nodes were measured after 8 weeks of infection. (**i–k**) Cell numbers recovered and percentage cell populations from lymph nodes. Representative images and H&E stained (**l**) BALB/c and (**m**) C57Bl/6 ears after 9 weeks and 8 weeks of infection, respectively. Results are the mean ± SEM of n = 5 mice/group and representative of two independent experiments (BALB/c) and n = 6 mice/group from one experiment (C57BL/6), where N denotes the necrotic ear lesions. Statistical analysis was performed defining differences to vehicle treated control mice as significant (*p < 0.05; **p < 0.01; ***p < 0.001).

To investigate the effect of a topical application of simvastatin on the progression of cutaneous leishmaniasis in mice, we used a previously established murine ear-model of *L. major* infection (1 × 10^3^)^19^. This model allowed us to generate practically accessible lesions on which the topical treatment could be applied. The ear model is particularly sensitive to the quantity of parasites inoculated, where resistant C57BL/6 mice have poorly detectable signs of infection at low doses^19^. Thus, we used a low dose of 1 × 10^3^ parasites for BALB/c only (Fig. 1) and a ten-fold higher dose of 1 × 10^4^ parasites for both BALB/c and C57BL/6 (Fig. 2).

With 1×10^3^ parasites infection in BALB/c mice, we found that our daily regimen of topical simvastatin treatment (Fig. 1a) resulted in visibly decreased ear swelling (Fig. 1b) and ulceration (Fig. 1g), which was further accompanied by reduced parasite burdens in the ear and cervical lymph nodes (LN) (Fig. 1c) after 10 weeks of infection. Interestingly, the treatment had no effect on the number of cells recruited to the draining LNs (Fig. 1d), nor was there any difference in the percentages of T and B-lymphocytes (Fig. 1e), or myeloid cells such as macrophages, dendritic cells and neutrophils in the LN (Fig. 1f).

Topical treatment of simvastatin in BALB/c mice infected with a 10-fold higher inoculum (1 × 10^4^) had almost identical results, with reduced lesion swelling (Fig. 2b) and ulceration (Fig. 2l). Furthermore, histological analysis using H&E staining revealed decreased tissue destruction in simvastatin-treated mice when compared to control animals (Fig. 2l). Again, treatment in BALB/c mice was also accompanied by reduced parasite loads in ear and draining cervical lymph nodes (Fig. 2c) and did not show any significant changes in cell numbers or percent immune cell recruitment (Fig. 2d–f). More interestingly, topical simvastatin was also able to show a protective effect in genetically resistant C57BL/6 mice (Fig. 2g), resulting in reduced parasite burden in lymph nodes (Fig. 2h) and no ulceration (Fig. 2m). Similar to BALB/c mice, no significant differences were found in cell numbers or percentages of immune cell populations recruited to the lymph nodes (Fig. 2i–k) however histological analysis using H&E staining revealed decreased tissue destruction in ear lesions of simvastatin-treated C57BL/6 mice (Fig. 2m). Together, these results demonstrate the potential use of simvastatin as a topical treatment for cutaneous leishmaniasis.

### Systemic simvastatin treatment increased protection against *L. major* infection in both susceptible BALB/c and resistant C57BL/6 mice

**Figure 3:**
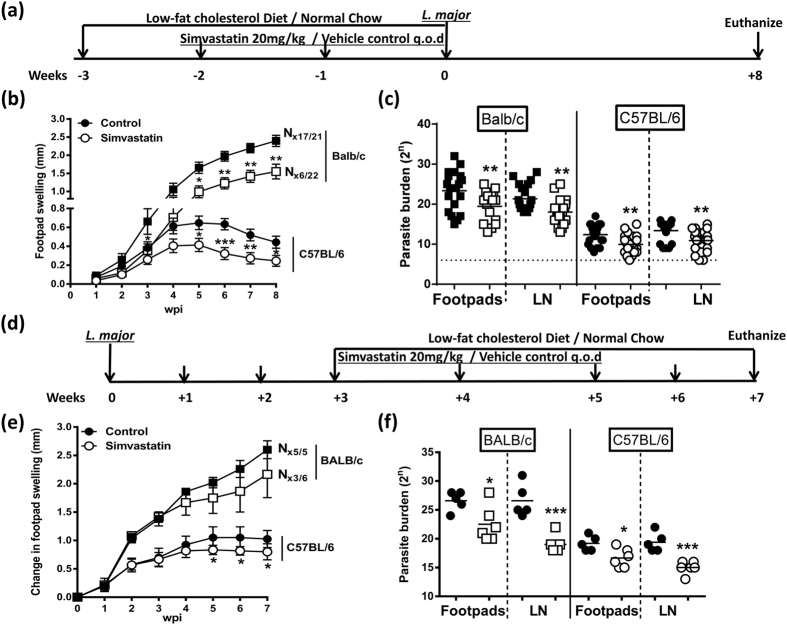
Systemic administration of simvastatin reduces the severity of subsequent *L. major* infection both in BALB/c and C57BL/6 mice. (**a**) Mice were fed a low-fat cholesterol diet (LFCD) or normal chow diet for three weeks. In the second and third weeks, mice treated every second day with intra-peritoneal injections of either simvastatin or vehicle control. At the end of treatment, mice were infected in the hind footpad with 2 × 10^6^ stationary phase metacyclic *L. major* LV39 parasites as shown in the layout. (**b**) Footpad swelling in BALB/c and C57BL/6 mice was measured to monitor disease progression at weekly intervals. (**c**) Parasite burdens in footpads and draining popliteal lymph nodes was determined by limiting dilution assay after 8 weeks of infection. Data represents the mean of four pooled independent experiments (n = 21–22 mice/group). (**d**) Mice were infected with *L. major* parasites as in (**a**), after 3 weeks of infection mice were treated with simvastatin for two weeks via intraperitoneal injections and fed on LFCD as shown in the layout. (**e**) Footpad swelling was monitored in BALB/c and C57BL/6 mice. (**f**) Parasite burdens were measured in footpads and popliteal lymph nodes after 7 weeks of infection. Data represented as mean ± SEM of n = 5–6 mice/group from one experiment, where N denotes the necrotic footpad lesions. Statistical analysis was performed defining differences to vehicle treated control mice as significant (*p < 0.05; **p < 0.01; ***p < 0.001).

**Figure 4:**
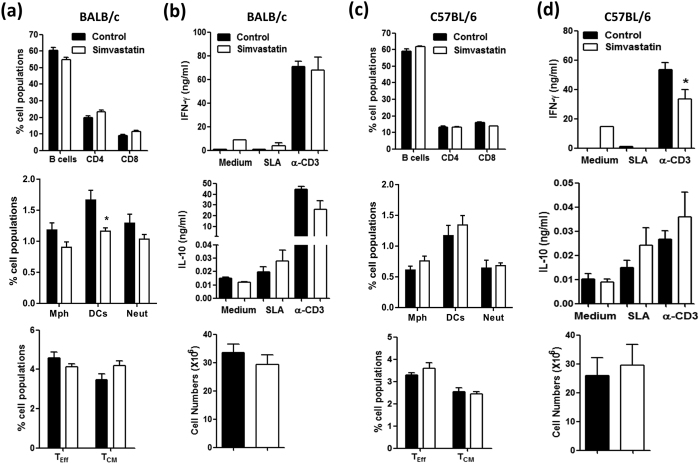
Simvastatin treatment has no effect on immune cell populations and cytokine production during *L. major* infection in both BALB/c and C57BL/6 mice. (**a**,**c**) Panels represents the percentage of immune cell populations (B cells, T cells, macrophages, DCs and neutrophils) and T effector and central memory cells recovered from lymph nodes in BALB/c **(a)** and C57BL/6 **(c)** mice following *L. major* LV39 infection. (**b**,**d**) Panels represents IFN-γ and IL-10 production in total lymph node cells (2 × 10^6^) re-stimulated *ex vivo* with Soluble *Leishmania* Antigen (SLA) and plate bound anti-CD3 by ELISA. Additionally, these panels show cell numbers recovered in lymph nodes of BALB/c and C57BL/6 mice after 8 weeks of infection. Results are the mean ± SEM of n = 5 mice/group and representative of two independent experiments. Statistical analysis was performed defining differences to vehicle treated control mice as significant (*p < 0.05).

We next examined whether the systemic administration of simvastatin could act as a prophylaxis or therapeutic against subsequent *L. major* (LV39, 2 × 10^6^) infection. To this end, simvastatin was administered at 20 mg/kg via intraperitoneal injections every other day for two weeks before inoculation in the hind footpad as shown in the layout (Fig. 3a). Similar to topical application, we observed that simvastatin treated BALB/c and C57BL/6 mice had significantly reduced footpad swelling, maintained up to 8 weeks of infection (Fig. 3b). Further, parasite loads in both footpads and draining popliteal lymph nodes were significantly reduced when compared to control mice (Fig. 3c). Next, we investigated the therapeutic potential of simvastatin by treating mice after 3 weeks of *L. major* infection as shown in the layout (Fig. 3d). We found no effect of simvastatin on footpad swelling in BALB/c mice however, slight differences were observed in C57BL/6 mice (Fig. 3e). Despite the slight differences in footpad swelling, interestingly post-treatment with simvastatin decreased the parasite burden in footpads and popliteal lymph nodes in both BALB/c and C57BL/6 mice (Fig. 3f). As in our previous results using topical simvastatin, percentages of cell recruitment and total cell numbers harvested from lymph nodes in both BALB/c (Fig. 4a,b) and C57BL/6 (Fig. 4c,d) mice were unaffected following pre-treatment. Also mirroring the topical treatment, we found no major differences in cytokine production such as IFN-γ and IL-10 between systemically simvastatin-treated and control in both BALB/c (Fig. 4b) and C57BL/6 (Fig. 4d) mice. This suggests that simvastatin treatment had no effect on the cytokine production during *L. major* infection. Similarly, serum SLA-specific antibody responses were unaffected during systemic simvastatin treatment in both BALB/c and C57BL/6 mice (data not shown). Taken together, these results suggest that systemic administration of simvastatin increases host protective immune responses against *L. major* infection in both BALB/c and C57BL/6 mice, and this enhanced immunity was not mediated by cellular recruitment to the draining lymph nodes or by T-cell cytokine production.

### Simvastatin reduces macrophage parasite burden by enhancing oxidative burst

**Figure 5:**
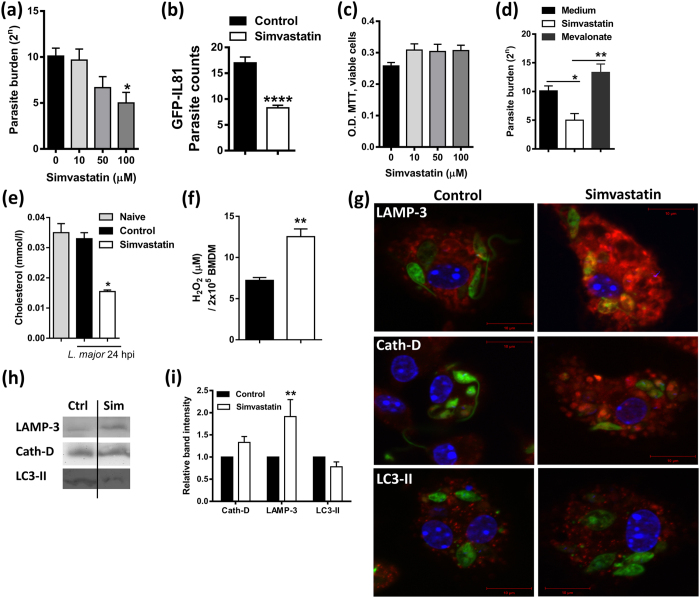
Simvastatin treatment in primary macrophages decreases parasite loads and enhances both hydrogen peroxide radical production and phagosome maturation. (**a,b**) Bone marrow-derived macrophages were treated with varying concentration of simvastatin before infection with *L. major* parasite (MOI: 10:1), parasite burden was assessed 24 hours post-infection by **(a)** limiting dilution assay and (**b**) number of GFP-expressing *L. major* (IL81) parasites. (**c**) Macrophages were treated with varying concentration of simvastatin to measure cell viability by MTT assay after 48 hours. (**d**) Mevalonate treatment rescues the inhibitory effect of simvastatin on parasite burdens in macrophages. (**e**) Simvastatin treatment reduces intracellular cholesterol levels in *L. major*-infected macrophages. (**f**) Simvastatin treatment increased the production of hydrogen peroxide (H_2_O_2_) in infected macrophages. (**g**) Confocal images of macrophages treated with simvastatin and infected with GFP-expressing IL81 *L. major parasites*. Phagosome and autophagy markers are indicated on the panels, IL81 *L. major* parasites are shown as green, LAMP-3, Cathepsin D and LC3-II are red and the DAPI stained nucleus is blue. (**h,i**) Western blot and densitometry showed significant increase in phagosome (LAMP-3) marker in total macrophage cell lysates upon *L. major* infection. Results are the mean ± SEM of triplicates and are representative of two independent experiments (*p < 0.05; **p < 0.01; ****p < 0.0001).

To investigate the cell intrinsic effect of simvastatin treatment on the internalisation and persistence of *L. major*, murine macrophages were pre-treated with simvastatin. Parasite growth determined at 24 hours post infection (Fig. 5a) showed a significantly reduced parasite burden in macrophages pre-treated with a concentration of 100 μM of simvastatin by limiting dilution assay. In contrast to the pre-treatment strategy, post-treatment of macrophages with simvastatin following *L. major* infection had no effect on parasite growth in comparison to control cells (data not shown). We confirmed pre-treatment findings by infecting macrophages with GFP-expressing *L. major* IL81 parasites and subsequently enumerating them in macrophages using fluorescent microscopy (Fig. 5b). The reduced parasitic growth in macrophages was not due to a cytotoxic effect of the drug, as shown by a viability MTT assay (Fig. 5c) nor due to effect on parasite uptake after 24 hours of infection (data not shown). Oppositely, macrophages cultured with the cholesterol-precursor, mevalonate, increased parasite growth (Fig. 5d), which suggested that the parasitotoxic effect of simvastatin was a result of its inhibitory action on the cholesterol biosynthesis pathway. Indeed, simvastatin treatment was associated with decreased cholesterol content in BMDMs when compared to control cell lysates (Fig. 5e). Moreover, simvastatin-treated BMDMs displayed significantly higher levels of hydrogen peroxide than control macrophages at 24 hours post infection (Fig. 5f). As it has been shown that hydrogen peroxide kills *Leishmania* parasites in wild type macrophages^20^, this finding highlights a potential mechanism for the anti-leishmanial effect of simvastatin. Thus, simvastatin-treated macrophages have reduced parasite burden, which may be due to simvastatin-dependent enhancement of host oxidative killing functions.

### Simvastatin increased phagosome maturation in *Leishmania*-infected macrophages

As an evasion mechanism, *Leishmania* parasites can block phagosome maturation^21^ and thereby promote its survival within macrophages. In addition, it has been reported that induction of host autophagy increases parasite loads in BALB/c mice but not in C57BL/6 mice^22^. We hypothesised that simvastatin promotes the maturation of phagosomes thereby increasing macrophage-mediated killing of the parasites. We analysed two phagosome markers (LAMP-3 and Cathepsin D) and a marker for autophagy (LC3-II) in macrophages following *L. major* (GFP-IL81) infection. Indeed, after 24 hours of infection, simvastatin treated macrophages exhibited an increase in the expression of LAMP-3 and Cathepsin D. However, simvastatin treatment had no effect on LC3-II induction, as demonstrated by qualitative confocal microscopy (Fig. 5g). Results from confocal microscopy were further confirmed by Western Blot analysis and densitometry, where LAMP-3 was increased, but LC3-II remained unaffected (Fig. 5h,i). Together, these results suggest that simvastatin treatment mediates phagosome maturation thereby increasing the clearance of *Leishmania* parasites in macrophages.

## Discussion

Here, we demonstrated that host cholesterol biosynthesis plays a role in the pathogenesis of cutaneous leishmaniasis. The inhibition of cholesterol biosynthesis by simvastatin, (which targets the host HMG-CoA reductase) reduced the growth of intracellular *Leishmania major* parasites in primary macrophages as well as in an *in vivo* mouse model (ear and footpad) of infection in both BALB/c and C57BL/6 mice.

In an effort to investigate the effect of simvastatin as a therapeutic agent at the site of infection, we explored a more practical topical administration of simvastatin directly on cutaneous ear lesions. Our results showed a significant improvement in presentation of the disease in both simvastatin-treated C57BL/6 and BALB/c mice. Local application of simvastatin has been used in the field of wound healing, where topical simvastatin promoted the resolution of *Staphylococcus aureus*-inoculated cutaneous wounds in mice^23^. A recent study built on this evidence, reporting that reduced inflammatory cytokines and bacterial burdens in the wounds caused by methicillin-resistant *S. aureus* (MRSA) in BALB/c mice, was due to the ability of simvastatin to suppress bacterial biofilm formation as well as the synthesis of proteins and MRSA toxins^24^. Another study highlighted the immunomodulatory role of statins in wound healing where the topical application of atorvastatin on traumatic lesions in rats (8 mm biopsy punch) resulted in accelerated tissue repair^25^. Consistence with these findings, topical simvastatin in our ear infection model of *L. major* also reduced lesion size and intra-lesion parasite burdens. However, more surprising and important was that topical treatment was also able to decrease parasite loads in draining cervical lymph nodes.

Besides, reduced severity of disease by topical application of simvastatin in ear infection model, we also found that systemic treatment with simvastatin before *L. major* infection (prophylactic) was protective in both the resistant C57BL/6 as well as the susceptible BALB/c mice as host-directed agent during footpad infection. The improved disease outcome in animals was independent of Th1 cytokines, type1 antibody production and host genetic background. Furthermore, simvastatin treatment after *L. major* infection (therapeutic) had no major effect on inflammatory response in footpads of the animals. Interestingly, this strategy also controlled parasite burdens in footpads and draining lymph nodes further suggesting that immunomodulatory function of simvastatin restricted parasite burdens at cellular level. In contrast to our findings, a study demonstrated that lovastatin treated mice had no significant difference in footpad swelling or parasite burdens when challenged with *L. major* at 5 weeks post infection^18^. This difference might be attributed to the use of activated lovastatin, being a less potent statin than simvastatin, an early time point analyzed and mice were fed on a normal chow diet.

Oppositely, the beneficial role of pravastatin was previously demonstrated in increased survival of *L. amazonensis*-infected susceptible BALB/c mice, but not in the survival of C57BL/6 mice after 50 weeks of infection^15^, indicating that the effect may depend on underlying immune and genetic biases within the host. The same authors reported that pravastatin treated BALB/c and C57BL/6 mice displayed reduced footpad swelling in *L. amazonensis* infection^16^ with pravastatin-treated peritoneal macrophages from BALB/c mice showing increased macrophage killing effector functions^16^. Since statins are known to act independently of their cholesterol-lowering properties^7^, the observed outcome of cutaneous leishmaniasis in this study might be due to the modulation of intermediates of the mevalonate pathway, which are targeted by simvastatin.

In terms of host cholesterol modulation in leishmaniasis, it appears from our findings and others that hypocholesterolemia induced by statins is important in decreasing the presentation of cutaneous form of disease in mice. In contrast, hypercholesterolemia induced either extrinsically (atherogenic/high fat diet) or intrinsically by deletion of apolipoprotein E gene (apoE^−/−^ mice), is crucial for increased host protection against visceral *L. donovani* infection in mice^17^. This suggests cholesterol plays a differential role in the pathogenesis of cutaneous and visceral forms of leishmaniasis.

Perhaps more interesting is that the observed reduction in parasite growth might be a direct effect of statins on the *L. major* HMG-CoA reductase^26^. Several other studies have reported a direct anti-fungal role for statins in extracellular culture^27^,^28^,^29^. In contrast, we observed no effect on extracellular growth of *L. major* promastigote parasites in presence of simvastatin at various concentrations tested (data not shown). Thus, the prophylactic and therapeutic effect of our treatment strategies might function by targeting HMG-CoA reductase in the host, which could disrupt the complex pathways of immune evasion that permit parasite survival and persistence.

Finally, an important mechanism of immune evasion by *Leishmania* parasites is their ability to suppress phagolysosome fusion, thereby creating a safe haven within macrophages to enable its persistence and growth^21^. We demonstrate a role for simvastatin in rescuing *Leishmania*-induced suppression of phagosome maturation (LAMP-3), thereby decreasing parasitic loads. Interestingly, in this study autophagy remained unaffected in *Leishmania*-infected macrophages, in contrast to *Mtb*-infected macrophages as we described previously^14^, suggesting that the simvastatin-mediated effect on autophagy may be pathogen-specific. This could also be due to the fact that autophagy (LC3-II) during *Leishmania* infection is dependent on the host genetic background^22^. Another well-known immune evasion strategy deployed by *Leishmania* parasites is to avoid microbicidal functions such as oxidative burst of peroxides^30^. Our finding demonstrated that simvastatin-mediate increased production of hydrogen peroxide upon infection, which would aid the clearance of intracellular *Leishmania* parasites.

In summary, our findings reveal the therapeutic and prophylactic potential of simvastatin in experimental murine models of cutaneous leishmaniasis. Our mechanistic studies add to the growing literature on the immunomodulatory actions of statins, providing an interesting avenue for adjunctive drug development in cutaneous leishmaniasis.

## Methods

### Mice

BALB/c and C57BL/6 mice (8–10 weeks) were maintained under specific-pathogen-free conditions in individually ventilated cages at the University of Cape Town, Animal Research Facility. All experiments were performed in accordance with the South African National Guidelines and University of Cape Town practice for laboratory animal procedures. The protocol (AEC: 012/033) was approved by the Animal Ethics Committee, Faculty of Health Sciences, University of Cape Town, Cape Town, South Africa.

### Reconstitution of simvastatin

Simvastatin (Sigma-Aldrich) was reconstituted in 1% DMSO with PBS at 10 mg/ml. Drug was then diluted 10 times in PBS to achieve 1 mg/ml concentration. In parallel, vehicle control was prepared without drug, which resulted in final concentration of 0.1% DMSO in PBS. During optimization in our previously published studies using simvastatin^13^,^14^, we found that vehicle control revealed no difference when compared to PBS, hence in the present study PBS group was not included.

### Topical application of simvastatin on the lesions caused by *Leishmania major* infection

Mice were infected subcutaneously in the dermis of the ear with 1 × 10^4^ (high dose) or 1 × 10^3^ (low dose) stationary phase *L. major* LV39 (MRHO/SV/59/P). *L. major* parasite strain was maintained by continuous passage in BALB/c mice as previously described^31^. On the first appearance of ear lesions, ears were treated daily for 6–8 weeks with topically applied simvastatin (20 μg/10 μl) or vehicle control (0.1% DMSO in PBS), immobilizing the mouse until the preparation was absorbed. Disease progression was assessed by measuring the ear lesion diameter weekly using a Vernier calliper.

### Systemic treatment of simvastatin before *L. major* infection

Mice were fed on low fat cholesterol diet (LFCD) or normal chow for three weeks. After one week, mice were intraperitoneally injected with 20 mg/kg of simvastatin every other day, for two weeks (whilst mice on normal chow were injected with vehicle control). After the 2-week treatment, anesthetized mice were subcutaneously infected in the left hind footpad with 2 × 10^6^ stationary phase *L. major* LV39 promastigotes. All mice were fed on normal chow after infection. Footpad swelling was measured weekly using a Mitutoyo micrometer gauge (Brütsch, Zürich, Switzerland). At 8 weeks post-infection, the infected footpads and draining popliteal lymph nodes were then collected for detection of viable parasites by limiting dilution assay^31^.

### Generation of bone marrow-derived macrophages (BMDMs) and *in vitro* infection with *L. major*

Bone marrow-derived macrophages (BMDMs) were generated from progenitor bone-marrow cells from 8 week old C57BL/6 mice as described previously^14^. BMDMs (2 × 10^5^) were then cultured in the presence of various concentrations of simvastatin or vehicle alone (0.004% DMSO final) overnight at 37 °C in 5% CO_2_ incubator. Cells were then infected with *L. major* LV39 parasites at an MOI of 10 parasites per macrophage for 24 hours at 37 °C followed by two washes with pre-warmed DMEM to remove non-internalized parasites. The detection of viable, phagocytosed parasites were determined by two-fold limiting dilution assay of lysed macrophages^32^.

### Cell viability assay

Cell viability of simvastatin-treated BMDMs was quantified using a colorimetric assay whereby the yellow tetrazolium salt MTT (3-(4,5-dimethylthiazolyl-2)-2,5-diphenyltetrazolium bromide) is reduced to a purple formazan by the mitochondrial enzymes of living cells. Macrophages (2 × 10^5^) were treated with simvastatin as above. 10 μl of MTT (5 mg/ml) was added and incubated, at 37 °C for 2 hours. This mixture was then solubilised in 0.04N HCL in isopropanol and incubated on a shaker for 30 min before the absorbance was read at 570 nm^14^.

### Measurements of macrophage cholesterol content and hydrogen peroxide

Simvastatin treated macrophages were infected with *L. major* (LV39) at an MOI of 10 for 24 hours or left uninfected. Cholesterol content was analysed in total macrophage cell lysates (3 × 10^6^), using a cholesterol assay kit (Bioassay system)^18^. Release of hydrogen peroxide (H_2_O_2_) from macrophages (2 × 10^5^) was measured using a colorimetric detection kit (Amplex Red Hydrogen peroxide assay kit, Molecular Probes)^16^.

### Fluorescence microscopy

Macrophages were seeded on coverslips and treated with 100 μM simvastatin overnight as above. Cells were then infected with green fluorescent protein (GFP) expressing- *L. major* (IL81) GFP-IL81 (MHOM/IL/81/FEBNI) at MOI of 10. After 24 hours, cells were washed and fixed in 4% paraformaldehyde followed by labelling of phagosome markers with fluorescent antibodies against Lysosome Associated Membrane Protein-3 (LAMP-3, Santa Cruz) and a lysosomal protease (Cathepsin D, Santa Cruz). Macrophages were also labelled for Light Chain 3-II (LC3-II, Santa Cruz), a marker for autophagy. These markers were then visualized by counter-stained with Alexa 546 antibodies (Molecular probes, Invitrogen) followed by nuclear stain (DAPI). Coverslips were then mounted using mowiol-containing anti-fade on glass slides. Images were captured under Carl Zeiss 510 confocal microscope and analysed using Zen Blue software as previously described^14^.

### Western Blot Analysis

SDS-PAGE and Western Blot analysis was performed as previously described^14^. Briefly, macrophages (3 × 10^6^) were treated simvastatin (100 μM) as above and then infected with *L. major* (LV39) at MOI of 10 parasites per macrophage. After 24 hours, cells were washed with pre-warmed medium to remove extracellular parasites. Macrophages were then lysed with ice-cold RIPA buffer containing protease inhibitor for 30 minutes at 4 °C. Total cell lysates were collected and analysed for protein content using BCA assay (ThermoFisher). Cell lysates with equilibrated protein volume (40 μg) were electrophoresed on 12% SDS-PAGE gel and then transferred to a nitrocellulose membrane (Sigma). The membrane was probed with anti-LAMP-3, Cathepsin D and LC3-II primary antibodies diluted (1:200) in blocking buffer at 4 °C overnight. Membrane was then incubated with an HRP-conjugated secondary antibody (1:10,000) for 1 hour at room temperature in blocking buffer. Immunoblots were developed using Super Signal West Dura substrate (Pierce).

### Statistical Analysis

All data were analysed using Graphpad Prism v 6.0, a unpaired student *t-*test (two-tailed with unequal variance). A ‘p’ value of less than 0.05 was considered significant.

## Additional Information

**How to cite this article**: Parihar, S. P. *et al.* Topical Simvastatin as Host-Directed Therapy against Severity of Cutaneous Leishmaniasis in Mice. *Sci. Rep.* **6**, 33458; doi: 10.1038/srep33458 (2016).
